# Adenosine A1 Receptor Deficiency Aggravates Extracellular Matrix Accumulation in Diabetic Nephropathy through Disturbance of Peritubular Microenvironment

**DOI:** 10.1155/2021/5584871

**Published:** 2021-10-11

**Authors:** Dongli Tian, Jiaying Li, Linfeng Zou, Min Lin, Xiaoxiao Shi, Yuting Hu, Jiaxin Lang, Lubin Xu, Wenling Ye, Xuemei Li, Limeng Chen

**Affiliations:** Department of Nephrology, State Key Laboratory of Complex Severe and Rare Diseases, Peking Union Medical College Hospital, Chinese Academy of Medical Science and Peking Union Medical College, Beijing 100730, China

## Abstract

**Background:**

We previously observed that adenosine A1 receptor (A1AR) had a protective role in proximal tubular megalin loss associated with albuminuria in diabetic nephropathy (DN). In this study, we aimed to explore the role of A1AR in the fibrosis progression of DN.

**Methods:**

We collected DN patients' samples and established a streptozotocin-induced diabetes model in wild-type (WT) and A1AR-deficient (A1AR^−/−^) mice. The location and expression of CD34, PDGFR*β*, and A1AR were detected in kidney tissue samples from DN patients by immunofluorescent and immunohistochemical staining. We also analyzed the expression of TGF*β*, collagen (I, III, and IV), *α*-SMA, and PDGFR*β* using immunohistochemistry in WT and A1AR^−/−^ mice. CD34 and podoplanin expression were analyzed by Western blotting and immunohistochemical staining in mice, respectively. Human renal proximal tubular epithelial cells (HK2) were cultured in medium containing high glucose and A1AR agonist as well as antagonist.

**Results:**

In DN patients, the expression of PDGFR*β* was higher with the loss of CD34. The location of PDGFR*β* and TGF*β* was near to each other. The A1AR, which was colocalized with CD34 partly, was also upregulated in DN patients. In WT-DN mice, obvious albuminuria and renal pathological leisure were observed. In A1AR^−/−^ DN mice, more severe renal tubular interstitial fibrosis and more extracellular matrix deposition were observed, with lower CD34 expression and pronounced increase of PDGFR*β*. In HK2 cells, high glucose stimulated the epithelial-mesenchymal transition (EMT) process, which was inhibited by A1AR agonist.

**Conclusion:**

A1AR played a critical role in protecting the tubulointerstitial fibrosis process in DN by regulation of the peritubular microenvironment.

## 1. Introduction

Diabetic nephropathy (DN), the leading cause of end-stage renal disease (ESRD) [1], manifests with a progressive increase in proteinuria, with the deposition of extracellular matrix (ECM) components and subsequent glomerulosclerosis and tubulointerstitial fibrosis. As the major pathological changes and the crucial role in the progression of DN [2], renal fibrosis is triggered by high glucose, which destroyed the stability of the renal peritubular microenvironment. The renal peritubular microenvironment is comprised of peritubular capillaries, renal tubular epithelial cells, and interstitium between them. During the progress of renal interstitial fibrosis of DN, epithelial-mesenchymal transition (EMT) in tubular epithelial cells is a crucial event [3], with the loss of cell-cell contact, dysfunction of tight junction, and myofibroblast generation [4].

Adenosine, a nucleoside that is a constituent of RNA and yields adenine and ribose on hydrolysis, could regulate cell function via the P1 purinoceptor family signaling [5]. The adenosine receptors, identified as adenosine A1, A2a, A2b, and A3, are integral membrane proteins widely distributed among vertebrates [5]. Previous studies revealed that A2a receptor (A2aAR) could attenuate the development of tubulointerstitial fibrosis and glomerulosclerosis in diabetic rats, as well as other renal fibrosis models [6–8]. A2B receptor (A2bAR) was responsible for a profibrotic and proinflammatory response in renal fibroblasts [9], either the adenosine A3 receptor (A3AR) [10]. The adenosine A1 receptor (A1AR) is widely studied in kidneys for its crucial role in tubule-glomerular feedback (TGF) [11] and the anti-inflammatory role in different acute kidney injury models, such as renal septic AKI and IR injury [12–15]. Our previous study in diabetic Akita mice (Ins2^+/-^) with A1AR ablation showed more prominent mesangial expansion and interstitial fibronectin staining than Akita mice with A1AR and wild-type control [16]. Recently, we also demonstrated that A1AR played a protective role in proximal tubular megalin loss in DN, in which the mechanism might associate with the caspase-1-related pyroptosis pathway [17]. However, little attention has been focused on the role of A1AR in the chronic fibrotic process of DN. Some studies have confirmed the EMT process in human renal proximal tubular epithelial cells (HK-2) when incubated in medium containing high glucose or transforming growth factor *β*1 (TGF*β*1) [18, 19], but without data on the role of A1AR in the EMT process of HK2 cells.

The injury of renal microvessels, consisting of glomerular capillaries and peritubular capillaries, can lead to ischemia and hypoxia of local tissue, which induces the activation of fibroblasts and the deposition of extracellular matrix components [20]. CD34, a marker of vascular endothelial cell, has been identified to be associated with vascular injury of DN [21]. A recent study suggested that A2BAR could ameliorate the pulmonary microvascular endothelial injury induced by lipopolysaccharide [22]. However, little attention has been focused on the effects of A1AR on microvascular injury-related fibrosis in DN.

The pericytes, which were important in maintaining vascular integrity and EPO production [23–25], could be activated by ischemia and hypoxia secondary to the loss of peritubular capillaries (PTC). The increase of PDGFR*β*, a marker of pericytes, demonstrated that the pericytes transit to myofibroblasts, leading to the process in fibrogenesis [26, 27] and the deposition of ECM [20]. A previous study revealed that activation of A1AR triggered contraction of pericytes [28], but the further relationship between A1AR and pericyte transition during fibrosis of DN was not elucidated.

In this study, we aimed to disclose the role of A1AR in the fibrosis process of DN. The EMT and ECM accumulation were observed in DN patients, established diabetes model in A1AR^−/−^ mice, and cultured HK2 cells treated with high glucose and A1AR agonist as well as antagonist. The renal peritubular microenvironment was evaluated preliminarily, including the tubular cells, peritubular capillaries, pericytes, and tight junction.

## 2. Materials and Methods

The reagents and antibodies are listed in Table S1.

### 2.1. Patients

Patients who were diagnosed as DN by biopsy were included in Peking Union Medical College Hospital (PUMCH) from January 2015 to December 2017. The control group included the patients (*n* = 14) with isolated microscopic hematuria diagnosed as a glomerular minor lesion (GML) without the foot fusion of podocytes. The study protocol was approved by the Institutional Ethics Committee at PUMCH (2014-2-18), and all subjects signed written informed consent.

### 2.2. Animals

Healthy male C57BL/6 mice weighing 18~22 g (age: 6~7 weeks) were acquired from Beijing Vital River Laboratory Animal Technology Company. Male A1AR-deficient (A1AR^−/−^) mice were presented by Professor Jurgen Schnermann from NIDDK of NIH (USA). All mice were kept in a specific pathogen-free (SPF) environment and adaptively fed for one week (ambient temperature 20~24°C, relative humidity 50%-55%, light cycle 12-12 hrs, and free drink and food) before the establishment of the diabetes model. The protocol of the animal experiment was approved by the PUMCH Institutional Ethics Committee of Animal Care and Use (ID: XHDW-2014-0024). All animal experiments were conducted following the national guidelines and the relevant national laws on the protection of animals.

### 2.3. Animal Models

Wild-type (WT) and A1AR^−/−^ (KO) mice, matched with age, weight, and blood glucose, were randomly assigned to three groups (6 mice in each group), including the wild-type control group (WT-control), the wild-type diabetes group (WT-DM), and the A1AR-deficient diabetes group (A1AR^−/–^DM). The type 1 diabetes mouse models were established by injecting STZ (Sigma, USA) (120 mg/kg, i.p.) dissolved in sodium citrate buffer (pH = 4.5) for two consecutive days, and the control mice were treated with sodium citrate buffer. Diabetes was confirmed with random blood glucose higher than 16.7 mmol/L, accompanied by polydipsia, polyphagia, polyuria, and emaciation. All mice were observed for 16 weeks before sacrifice.

### 2.4. Cell Culture and Treatment

Human renal proximal tubular epithelial cell lines (HK2) were obtained from the Cell Resource Center of the Chinese Academy of Medical Sciences. HK2 cells were cultured in DMEM/F12 medium (Gibco, USA) with 10% fetal bovine serum (FBS, Gibco, USA) and 1% penicillin-streptomycin solution. The cells were confirmed as HK2 by cellular morphological characteristic identification under phase-contrast microscopy and the cellular specific markers (cytokeratin 18 and megalin) with immunofluorescence staining. HK2 cells were in a serum-free medium for 12 hrs before treatment, then exposed to low glucose (5 mmol/L), high glucose (25 mmol/L), and high mannitol (25 mmol/L), respectively, for 24 and 72 hrs. Appropriate concentrations of A1AR agonist (CCPA, 0.1 *μ*mol/L, Sigma, USA) and antagonist (DPCPX, 1 *μ*mol/L, Sigma, USA) were chosen by CCK8 and LDH assay. After 24 hr coculturing with high glucose and CCPA or DPCPX, HK2 cells were harvested for Western blotting analysis.

### 2.5. Sample Collection

The body weight of each mouse was recorded weekly. Blood glucose was measured by tail venipuncture with One Touch Ultra Test Strips (Johnson & Johnson, USA). 24-hour urine was collected in a MMC100 metabolic cage (Hatteras, USA). After modeling for 16 weeks, mice were sacrificed by anesthesia and perfused with 0.9% precooled saline from the heart and aorta, and the kidneys were rapidly dissected. The renal cortex and medulla were separated, snap-frozen in liquid nitrogen, and stored at -80°C for the next experiment.

### 2.6. Histology

The three-*μ*m-thick paraffin-embedded kidney tissue sections were stained with hematoxylin and eosin (HE) and Masson's trichrome for light microscopy (Olympus, Japan). A transmission electron microscope was used to distinguish the detachment between pericytes and endothelial cells (JEM-1400plus, Japan).

### 2.7. Immunohistochemistry and Immunofluorescence

Three-*μ*m sections cut from paraffin-embedded tissue were deparaffinized, rehydrated, and antigen retrieved, then incubated with the primary antibody overnight at 4°C. Secondary antibodies were HRP-conjugated goat anti-rabbit (ImmunoReagents, USA). All section images were viewed with the microscope (Eclipse 80i; Nikon, Japan) with a digital photograph camera (DS-U1; Nikon, Japan). IHC staining was analyzed using ImagePro Plus 6.0 by calculating the percentage of the positive area. Scoring was evaluated by a “blinded” investigator on coded slides. At least eight fields were selected randomly to cover the majority of the cortex per specimen for photodocumentation. Immunofluorescent (IF) staining was performed on serial sections of patients with biopsy-confirmed DN using standard methods. Secondary antibodies were DyLight 488 AffiniPure Goat Anti-Rabbit IgG (H+L) or DyLight 594 AffiniPure Goat Anti-Mouse IgG (H+L) (Abbkine, USA). The micrographs of immunofluorescent stains were captured by confocal laser microscopy (Leica, Germany).

### 2.8. Western Blotting Analysis

Total protein was extracted from the renal cortex and HK2 cells for immunoblotting analysis with the primary antibodies for A1AR, CD34, collagen I, collagen III, TGF*β*, vimentin, and occludin. *β*-actin was used as the internal reference protein. The secondary antibody was HRP-conjugated goat anti-rabbit; then, an enhanced chemiluminescence detection system (Tanon 5200, China) was used to detect the immunoblotting signals. Quantification was performed by ImageJ Microsoft (NIH, USA).

### 2.9. Statistical Analysis

The unpaired *t*-test (two-tailed) was used to compare the difference between the two groups. One-way ANOVA with Dunnett's multiple comparison test was used for comparison among the multiple groups. *P* < 0.05 was considered statistically significant. The values were presented as the mean ± SEM. All statistical analysis was performed by GraphPad Prism 7 software.

## 3. Results

### 3.1. Peritubular Capillary Loss with Activation of PDGFR*β* and TGF*β* in DN Patients

**Figure 1:**
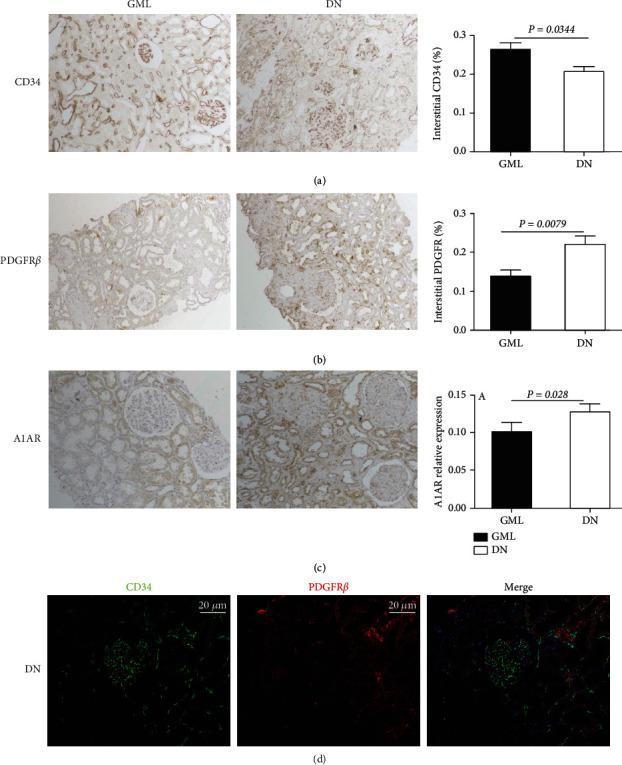
Immunohistochemical staining showed tubulointerstitial CD34 loss with the accumulation of PDGFR*β* and A1AR in patients with GML and DN. (a–c) The expression of CD34, PDGFR*β*, and A1AR in GML and DN patients was measured by semiquantitative analysis of immunohistochemical staining. (d) The costaining of CD34 and PDGFR*β* in DN patients. Data were presented as the mean ± SEM (*n* = 6~10 per group). ^∗^*P* < 0.05, ^#^*P* < 0.05. Original magnification = 200x. Bar width: 50 *μ*m. GML: glomerular minor lesion; DN: diabetic nephropathy.

**Figure 2:**
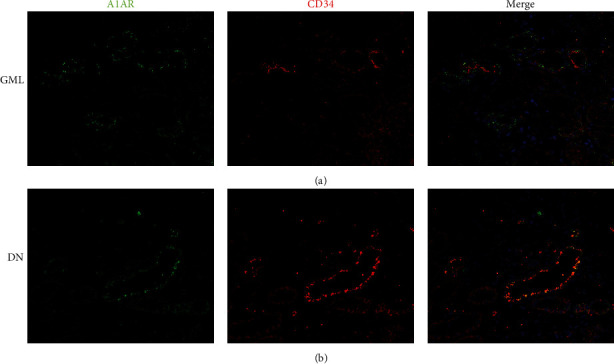
Costaining of A1AR and CD34 in kidney tissues of GML and DN patients by immunofluorescent staining. (a) A1AR was mainly expressed on the renal proximal tubular cell membrane, while CD34 was expressed on microvascular cells of GML patients. (b) A1AR and CD34 were colocated at the brush border of proximal tubule cells and peritubular capillaries in DN patients by immunofluorescent staining. Original magnification = 400x. Green: A1AR; red: CD34; blue: DAPI; DAPI: 4′,6-diamidino-2-phenylindole. Bar width: 50 *μ*m.

Immunohistochemical semiquantitative analysis showed that the expression of CD34 was lower in DN patients (Figure 1(a)), as well as the higher expression of PDGFR*β* in DN patients, compared to GML patients (Figures 1(b) and 1(c)). The location of CD34 and PDGFR*β* was adjacent to each other (Figure 1(d)). Moreover, A1AR and CD34 were colocated at the brush border of proximal tubule cells and peritubular capillaries in DN patients by immunofluorescent staining, while not in GML patients (Figure 2(b)).

### 3.2. A1AR Deletion Exacerbated Fibrosis Process and Vascular Endothelial Cell Injury

**Figure 3:**
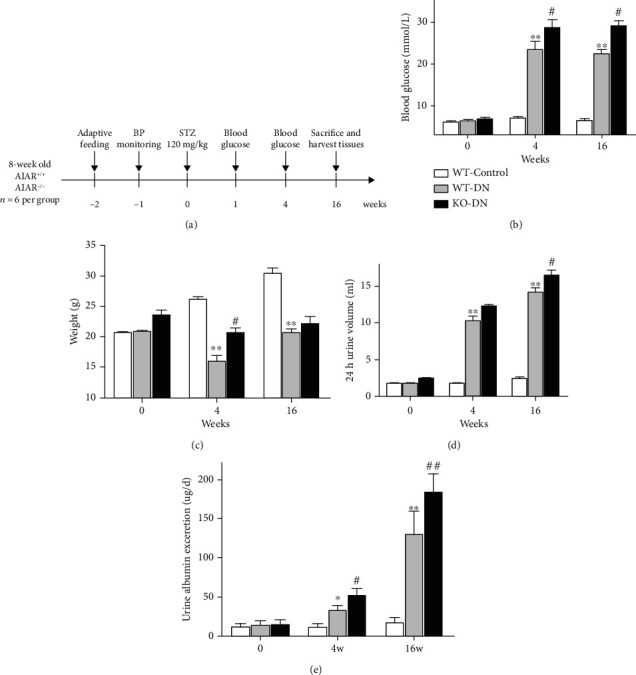
Basic physiological index of the diabetes mouse model at the age of 4 and 16 weeks. (a) The flow chart of the mouse model in this study. (b–e) The changes in blood glucose, weight, 24 h urine volume, and urine albumin excretion on day 0, day 28, and day 112 in three groups. Blood glucose ≥ 16.7 mmol/L was defined as diabetes. *N* = 6 per group. Data was shown as the mean ± SEM. WT-control vs. WT-DN: ^∗^*P* < 0.05 and ^∗∗^*P* < 0.001; WT-DN vs. KO-DN: ^#^*P* < 0.05 and ^##^*P* < 0.001.

We successfully established type 1 diabetes mouse models induced by STZ. The experiment flow chart and basic physiological index of mice in 3 groups were shown (Figure 3). The blood glucose level was significantly higher in both WT-DN and the KO-DN mice than WT-control mice, while more pronounced in the KO-DN group (Figure 3(b)). Both diabetic groups had markedly higher urine volume and 24 hr urinary albumin excretion, as well as lower body weight (Figures 3(c)–3(e)). The 24 hr urinary albumin excretion was much more profoundly elevated in KO-DN mice than in the WT-DN group (70.8 ± 4.1 vs. 32.0 ± 2.9, *P* = 0.0015 at week 4 and 183.8 ± 9.7 vs. 129.4 ± 12.2, *P* = 0.0008 at week 16).

**Figure 4:**
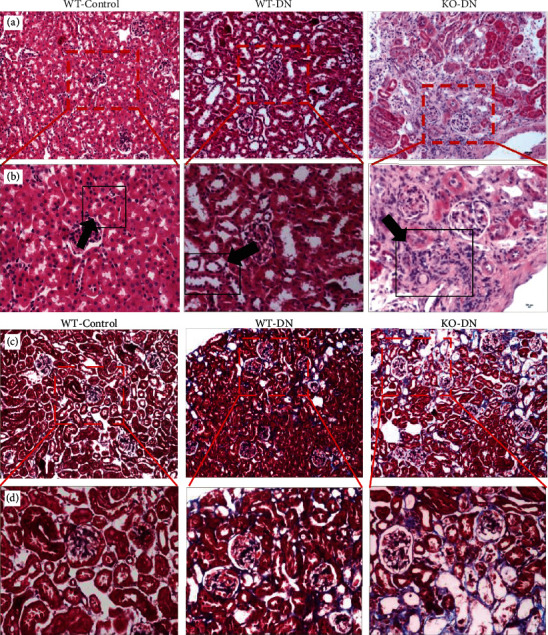
Representative pathological images by HE and Masson staining at 16 weeks: (a, b) HE staining; (c, d) Masson staining. The WT-DN group showed the disappearance of the normal tubular back-to-back structure with focal interstitial fibrosis compared to WT-control. The KO-DN group showed more severe tubulointerstitial fibrosis than that in the WT-DN group. (b, d) (200x) magnifications of the box in (a, c) (100x). *N* = 6 per group. Abbreviations: HE: hematoxylin and eosin.

**Figure 5:**
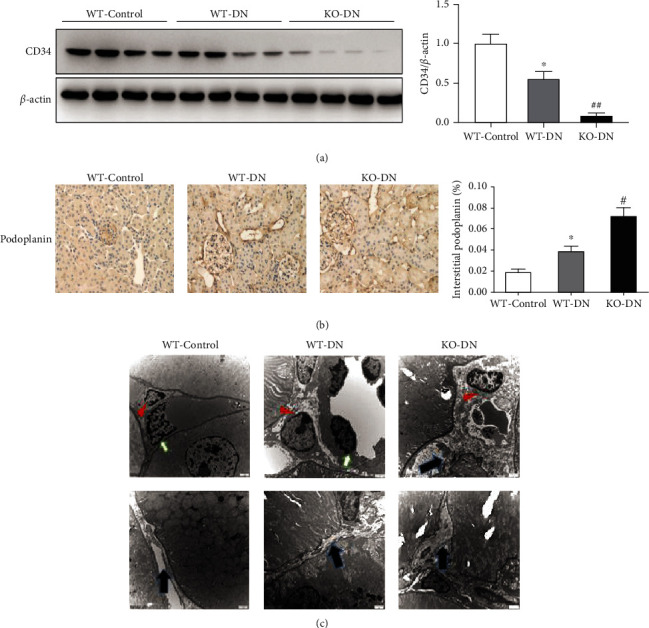
A1AR deletion accelerated renal vascular endothelial cell loss and peritubular microenvironment disorder in DN mice at 16 weeks. (a) CD34 expression by Western blotting. Compared to the WT-control group, CD34 expression was decreased in the WT-DN group, with a further decrease in the KO-DN group. (b) The expression of podoplanin by semiquantitative analysis of immunohistochemical staining in the WT-control, WT-DN, and KO-DN groups. (c) The detachment between pericytes and endothelial cells, with more collagen deposition. Red arrow: pericyte; white arrow: endothelial cells; blue arrow: collagen deposition. Data was shown as the mean ± SEM. *N* = 6 per group. The WT-control vs. WT-DN: ^∗^*P* < 0.05 and ^∗∗^*P* < 0.001; WT-DN vs. KO-DN: ^#^*P* < 0.05 and ^##^*P* < 0.001.

The HE and Masson staining (Figures 4(a)–4(f)) of WT-DN showed the disappearance of the normal tubular back-to-back structure with focal interstitial fibrosis at week 16, compared to WT-control, while KO-DN mice presented with more severe tubulointerstitial fibrosis than that in WT-DN mice. In the tubular interstitial, Western blotting showed that the CD34 expression was consecutively decreased in the order of WT-control, WT-DN, and KO-DN mice (Figure 5(a)), while the podoplanin expression was consecutively increased in these 3 groups (Figure 5(b)) by immunohistochemical staining.

**Figure 6:**
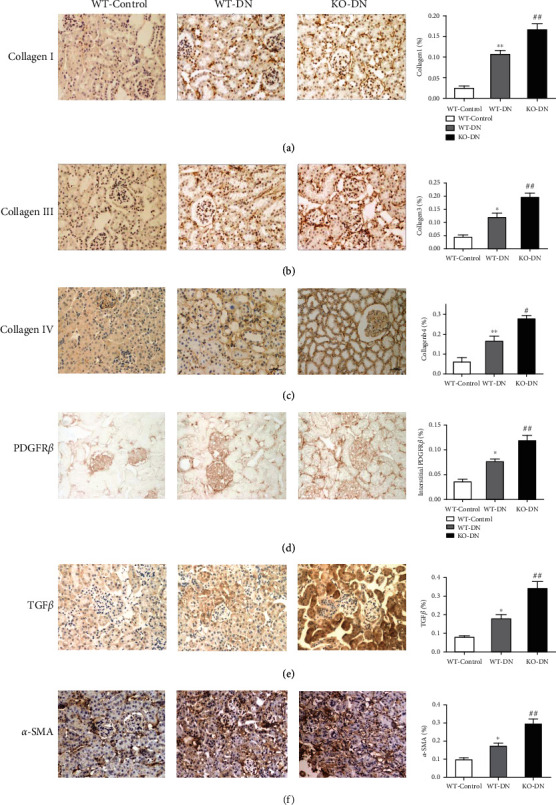
A1AR deletion aggravated interstitial collagen deposition, interstitial fibrosis, and activation of PDGFR*β* in DN mice. (a–f) The expression of collagen I, collagen III, collagen IV, PDGFR*β*, TGF*β*, and *α*-SMA by semiquantitative analysis of immunohistochemical staining. Compared to the WT-control group, the WT-DN group showed increased expression of all markers, while the increase was more overt in the KO-DN group. *N* = 6 per group. Data was shown as the mean ± SEM. The WT-control vs. the WT-DN: ^∗^*P* < 0.05 and ^∗∗^*P* < 0.001; WT-DN vs. KO-DN: ^#^*P* < 0.05 and ^##^*P* < 0.001. Original magnification = 200x.

A semiquantitative analysis showed that collagen (I, III, and IV), TGF*β*, and *α*-SMA expression was increased in WT-DN compared to WT-control mice, while KO-DN mice showed a more prominent elevation than WT-DN mice (Figures 6(a)–6(c), 6(e), and 6(f)). Besides, the expression of PDGFR*β* presented the same changing trend as fibrosis markers in 3 groups of mice (Figure 6(d)). We also observed that the detachment between pericytes and endothelial cells became much severe in KO-DN, compared to WT-DN mice by the electron microscope (Figure 5(c)).

### 3.3. The Protective Role of A1AR in the EMT Process In Vitro

**Figure 7:**
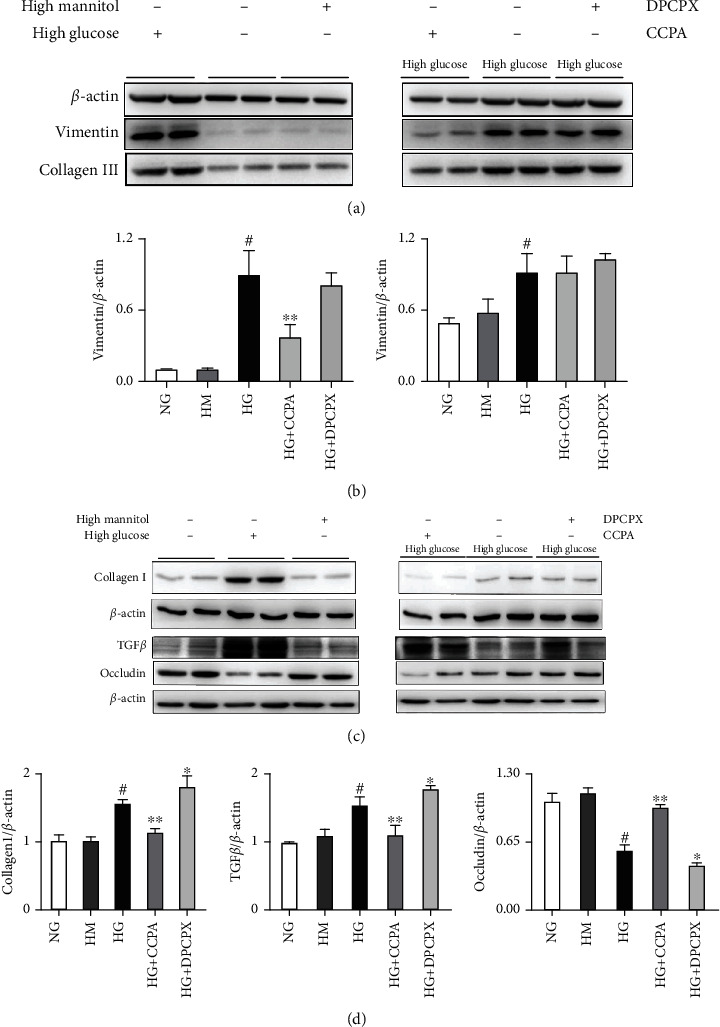
The role of A1AR agonist CCPA and antagonist DPCPX in EMT in HK2 cell cultured with high glucose. (a, b) Western blotting quantitative analysis for vimentin and collagen III of HKC cells cocultured with normal glucose, high glucose, high mannitol, and high glucose with CCPA and DPCPX. (c, d) Western blotting quantitative analysis for collagen I, TGF*β*, and occludin of HKC cells cocultured with normal glucose, high glucose, high mannitol, and high glucose with CCPA and DPCPX. Data are shown as the mean ± SEM; ^#^*P* < 0.05 HG vs. NG, ^∗^*P* < 0.05 HG vs. HG+DPCPX, and ^∗∗^*P* < 0.001 HG vs. HG+CCPA. Abbreviations: CCPA: A1AR agonist; DPCPX: A1AR antagonist.

In HK2 cells cultured with high glucose medium, the expression of mesenchymal markers, including collagen 1 and vimentin, was increased compared to the normal glucose and high mannitol groups (Figures 7(a) and 7(c)). Furthermore, adding A1AR antagonist (DPCPX) to high glucose medium increased collagen 1 and vimentin expression, while CCPA (A1AR agonist) inhibited the expression of them (Figures 7(a) and 7(c)). Moreover, the loss of occludin was observed in the high glucose groups and more obvious by DPCPX but abolished by CCPA (Figures 7(a) and 7(c)).

## 4. Discussion

A1AR is widely studied in kidneys for its key role in tubule-glomerular feedback (TGF). In this study, we first clarified the protective effects of A1AR on fibrosis progression in DN. In A1AR-deficient mice, the aggravated interstitial fibrosis is accompanied by the loss of peritubular capillaries, EMT of tubular cells, and the activation of pericyte, while the process could be abolished by A1AR agonist and aggravated with A1AR antagonist in HK2 cells. TGF*β* upregulation and tight junction dysfunction were also involved in the ECM accumulation process.

**Figure 8:**
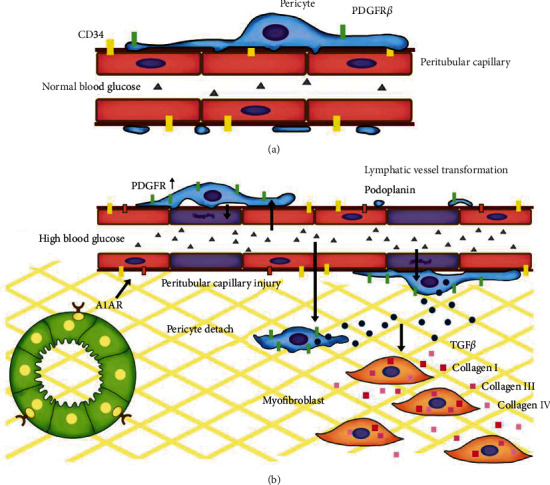
Microvascular pericytes in normal and injured kidneys. (a) In a healthy kidney, the pericytes of microvascular expressed PDGFR*β* closely contact with endothelial cells, maintaining proper endothelial function and microvascular integrity. (b) In the diabetic kidney, hyperglycemia activates microvascular pericytes to detach from the endothelial cells. Then, pericytes differentiated to myofibroblasts and migrated into the interstitium to produce large amounts of collagen, inducing pathologic extracellular matrix deposition. Consistent injury leads to unstable vasculature, capillary loss, interstitial matrix expansion, and tubular interstitial fibrosis. A1AR, widely distributed in the renal proximal tubule, interstitial, and vascular endothelial cells, might play a protective role in this procedure by inhibiting microvascular pericyte transformation and vascular loss. Abbreviations: PDGFR: platelet-derived growth factor receptor; TGF*β*: transforming growth factor *β*; A1AR: A1 adenosine receptor.

First, we proved that the fibrosis of DN was triggered by the loss of integrity in the peritubular microenvironment, which was composed of peritubular capillary endothelial cell, pericyte, interstitial matrix, renal proximal tubule, and lymphatic endothelial (Figure 8). The loss of peritubular capillaries, indicated by decreased CD34 expression, was an independent predictor of renal fibrosis in DN patients and animal models. Since CD34 is positive in both vessel endothelium and lymphatic endothelium, we excluded the injury of lymphatic endothelia [29] by podoplanin staining. In contrast to the renal blood vessel impairment, lymphatic vessel proliferation was obvious in DN mice, which was also the risk factor for tubulointerstitial fibrosis [29–31]. Besides the ischemia, poor waste drainage also promotes fibrosis further. The compensation for lymphatic vessel proliferation serves as export of interstitial fluid, inflammatory cells, and cytokines, but they might not function well under this condition [32]. Pericytes were activated by detaching from the abnormal vascular endothelial cells, which is consistent with the previous report in obstructive fibrosis of the kidney [27]. The pericyte differentiation is a primary source of the myofibroblast [27, 33, 34], which became a key step in producing the pathogenic collagen [35]. The increased expression of interstitial PDGFR*β* was used to describe the distribution of pericytes and myofibroblasts [36, 37] and could attenuate the renal fibrosis by blocking it [38].

The epithelial-mesenchymal transition (EMT) and secretion of TGF*β* were observed in HK2 cells stimulated by high glucose. EMT in tubular epithelial cells is a crucial event in the progression of renal interstitial fibrosis of DN, which is considered another source of myofibroblast generation [4]. This transition is characterized by the loss of cell-cell contact, identified by the decrease of integral membrane proteins, occludin, and claudins. Moreover, the upregulation of mesenchymal markers, including fibronectin, vimentin, and collagen I, is another character of EMT [19]. In our study, the EMT process and the expression of TGF*β* were alleviated when added A1AR agonist into the high glucose culture medium. Thus, these findings indicate the direct protective role of A1AR in EMT in vitro, which is an important process of renal interstitial fibrosis in DN.

We firstly clarified the protective effects of A1AR on the fibrosis progress of DN in A1AR-deficient DN mice and in vitro HK2 cells with A1AR antagonist or agonist. In this study and our previous data, both the diabetic Akita mice (Ins2^+/-^) with A1AR ablation [16] and A1AR-deficient STZ mice showed more prominent mesangial expansion and interstitial fibrosis. The role of A1AR in fibrosis was controversial [39–41] in the unilateral ureteral obstruction rat model of renal fibrosis. The A1AR mRNA level was increased significantly on day 5 [42], while it failed to observe significant variation in the A1AR mRNA level at week 2 and week 4 [43]. In this study, we confirmed the biphasic change of A1AR protein in WT-DN mice that A1AR initially elevated at week 4 but decreased at week 16 in the time-dependent fibrosis process diabetic mouse model.

Since the A1AR is expressed at the brush border of PTC, with the progress of the EMT and ECM accumulation, the A1AR loss might be secondary to the dysfunction of PTC. Besides, we observed more obvious peritubular capillary loss, pericyte transformation with PDGFR*β* expression, and more pronounced fibrosis in KO-DN mice. Moreover, A1AR could stimulate cell proliferation and promote wound healing in the EA.hy926 endothelial cells [44]. Together with our observation that CD34 and A1AR were adjacent to each other in the renal peritubular microenvironment in DN patients (Figure 2), it is a reasonable assumption that A1AR might attenuate renal fibrosis by protecting vascular endothelial cells in DN.

Although we firstly confirmed the direct protective role of A1AR in fibrosis of DN and EMT in HK-2 cells, there are some limitations. Because there is no mature technique to isolate renal pericytes successfully up to now, we cannot carry out the experiments in vitro to elaborate on the exact mechanism of the process by A1AR activation. Although we have proved that A1AR played a protective effect on megalin loss by inhibiting the pyroptosis-related caspase-1/IL-18 signaling in DN [17], to provide a more theoretical basis for A1AR agonist treatment of DN in the future, more experiments in vitro are needed to clarify the direct relationship between A1AR and peritubular microenvironment.

## 5. Conclusions

In summary, our study suggested that A1AR plays a protective role in renal fibrosis progression of DN in keeping the integrity of the tubular microenvironment. These findings suggest that the activation of A1AR may be a potential therapeutic strategy against DN.

## Data Availability

The data used to support the findings of this study are included within the article, and the data about reagents and antibodies used to support the findings of this study are included within the supplementary information file.

## Supplementary Materials

Supplementary Materials Reagents and antibodies used to support the findings of this study were included within the supplementary file.
