# Supplementary material for: Adenosine A1 Receptor Deficiency Aggravates Extracellular Matrix Accumulation in Diabetic Nephropathy through Disturbance of Peritubular Microenvironment
Source: J Diabetes Res. 2021 Oct 11;2021:5584871. doi: 10.1155/2021/5584871 (PMC8523293; doi:10.1155/2021/5584871)
Supplement: Supplementary Materials — Reagents and antibodies used to support the findings of this study were included within the supplementary file. [file 5584871.f1.docx]

**Table S1. Reagents and antibodies used in this study**

| **Reagent** | [**Manufacturers**](file:///C:/Program%2520Files%2520(x86)/Youdao/Dict/7.5.2.0/resultui/dict/%3fkeyword=manufacturers) | **Product code** | **Application(**[**dilution)**](file:///C:/Program%2520Files%2520(x86)/Youdao/Dict/7.5.2.0/resultui/dict/%3fkeyword=dilution) |
| --- | --- | --- | --- |
| STZ | Sigma (USA) | S0130 | IP (120mg/kg) |
| **Primary antibody** |  |  |  |
| CD34 | Abcam (UK) | ab81289 | WB (1:1000) |
| Collagen1 | Abcam (UK) | ab34710 | IHC (1:200) |
| Collagen3 | Abcam (UK) | ab7778 | IHC (1:200) |
| Collagen4 | Abcam (UK) | ab6586 | IHC (1:200) |
| A1AR | Abcam (UK) | ab82477 | WB (1:800) |
| PDGFR-β | Abcam (UK) | Ab91066 | WB (1:500) |
| Podoplanin | Abcam (UK) | ab10288 | IHC (1:100) |
| TGFβ | Abcam (UK) | ab92486 | IHC (1:100) |
| α-SMA | Abcam (UK) | Ab124964 | IHC (1:1000) |
| β-actin  Vimentin  Occludin | Abcam (UK)  Abcam (UK)  Abcam (UK) | ab8226  ab92547  ab216327 | WB (1:2000)  WB (1:2000)  WB (1:1000) |
| **Secondary antibody** |  |  |  |
| Goat anti-Rabbit | ZSGB (China) | PV9001 | IHC |
| Goat anti-Mouse | ZSGB (China) | PV9002 | IHC |
| Goat anti-Rabbit | Abkine (USA) | [A23210](http://www.abbkine.cn/product/a23210/) | IF (1:200) |
| Goat anti-Mouse | Abkine (USA) | A23410 | IF (1:200) |
| Goat anti-Rabbit | Abcam (USA) | [A21020](http://www.abbkine.cn/product/a21020/) | WB (1:1000) |

[**Abbreviation**](file:///C:/Program%2520Files%2520(x86)/Youdao/Dict/8.3.0.0/resultui/html/index.html#/javascript:;)**:** STZ: [Streptozotocin](file:///C:/Program%2520Files%2520(x86)/Youdao/Dict/7.5.2.0/resultui/dict/javascript:;), A1AR: A1 adenosine receptor, IP: Intraperitoneal injection, IHC: [Immunohistochemistry](file:///C:/Program%2520Files%2520(x86)/Youdao/Dict/7.5.2.0/resultui/dict/javascript:;), IF: [Immunofluorescence](file:///C:/Program%2520Files%2520(x86)/Youdao/Dict/7.5.2.0/resultui/dict/javascript:;), WB: Western blot.
